# Optimizing genomic DNA extraction from long-term preserved formalin-fixed and paraffin-embedded lung cancer and lymph node tissues

**DOI:** 10.1590/1414-431X2024e14095

**Published:** 2024-11-25

**Authors:** C.S. Faria, C.M. Baldavira, F.R.R. Mangone, M.E.M. Agati, L.D. Kulikowski, M.A. Nagai, E.C.T. do Nascimento, E.S. de Mello, V.L. Capelozzi, L. Antonangelo

**Affiliations:** 1 Laboratório de Investigação Médica, Hospital das Clínicas, Faculdade de Medicina, Universidade de São Paulo, São Paulo, SP, Brasil; 2 Laboratório de Histomorfometria e Genômica Pulmonar, Departamento de Patologia, Faculdade de Medicina, Universidade de São Paulo, São Paulo, SP, Brasil; 3 Laboratório de Genética Molecular, Centro de Pesquisa Translacional em Oncologia, Centro de Estudos e Tecnologias Convergentes para Oncologia de Precisão, Instituto do Câncer do Estado de São Paulo, Departamento de Radiologia e Oncologia, Faculdade de Medicina, Universidade de São Paulo, São Paulo, SP, Brasil; 4 Departamento de Patologia, Hospital das Clínicas, Faculdade de Medicina, Universidade de São Paulo, São Paulo, SP, Brasil; 5 Laboratório de Patologia do Instituto do Câncer do Estado de São Paulo, Departamento de Patologia, Faculdade de Medicina, Universidade de São Paulo, São Paulo, SP, Brasil; 6 Divisão de Patologia Clínica, Departamento de Patologia, Hospital das Clínicas, Faculdade de Medicina, Universidade de São Paulo, São Paulo, SP, Brasil

**Keywords:** Paraffin-embedded tissues, Slide scrapings, Genomic DNA extraction, Lung adenocarcinoma, Mediastinal lymph nodes

## Abstract

Personalized therapy in lung cancer (LC) has revolutionized routine histopathology and cytopathology, emphasizing the importance of obtaining adequate material for molecular studies to support oncological decisions. Adaptations of cytologic sample preparations offer benefits for molecular testing, yet their potential remains underutilized. A significant number of LC cases is identified through specimens of aspiration or exfoliative cytology. Improving screening approaches and optimizing tissue utilization for biomarker research are crucial for effective LC management. The utilization of formalin-fixed, paraffin-embedded (FFPE) tumor tissues has become standard practice in clinical and epidemiological genetic research. However, current techniques require not only a standardized sample fixation and storage but also sufficient genetic material to yield reliable results. In this study, we utilized the Qiagen GeneRead^®^ DNA FFPE kit with an adapted protocol for two extraction methods: one involved cutting FFPE blocks and the other involved scraping tissue from slides used for histochemical and cytological analysis. Our findings emphasized the importance of increasing the number of FFPE sections, heat deparaffinization, and adjusting proteinase K digestion time to enhance genomic DNA (gDNA) yields. Notably, scraping tissue from slides yielded superior results compared to the standard FFPE protocol. A median of 2.82 and 4.34 DNA yields for tumor and lymph node, respectively, were obtained. Our results demonstrated the feasibility of this adapted protocol for gDNA extraction in clinical and epidemiological studies. We recommend scraping tissue from slides as a reliable source of gDNA and suggest fine-tuning proteinase K digestion time and heat exposure based on the input tissue volume.

## Introduction

Tissue specimens utilized for oncological diagnostics are predominantly formalin-fixed and paraffin-embedded (FFPE), offering numerous benefits. However, the integrity of the obtained nucleic acids is often compromised, posing a challenge for subsequent molecular analyses. Achieving high-yield and high-integrity DNA extraction from a limited volume of tumor tissue is a critical pre-analytical procedure in specimen-based cancer genomic investigations (1-3). Fresh frozen specimens are ideal for yielding nucleic acids with superior integrity; however, they may be unavailable for many patients due to the challenges in standardized fixation requirements and processing time. In contrast, formalin-fixed paraffin-embedded tissue (FFPE) tumor specimens are widely available, even from patients diagnosed years before. Therefore, due to tissue fixation and prolonged storage, the damage and fragmentation of nucleic acids in archived FFPE samples present significant challenges for genetic studies. Nevertheless, technology advancements in recent years facilitated the utilization of DNA samples derived from FFPE tissues for next-generation sequencing (NGS), among other applications (4,5). Despite the expanded range of analyses that are possible with nucleic acids extracted from FFPE tissue, there is still a need for improvement in standard operating procedures for gDNA isolation from old tissue blocks.

There are numerous commercially available nucleic acid extraction kits, some specifically designed for DNA extraction, with previous studies assessing and comparing their performance for FFPE tumor tissues (6-10). Outstanding results have been achieved with FFPE tissue stored for up to four decades (9), with another study concluding that the Qiagen GeneRead^®^ DNA FFPE Kit demonstrated the best performance for FFPE tissues (7). These previous studies aimed to identify suitable extraction methods and were often conducted with a small number of samples and limited tissue portions, simultaneously employing multiple kits for comparison. Consequently, how these extraction methods perform in real-world investigative practices remains to be evaluated. Moreover, considering the variable quality of FFPE samples, it is essential to evaluate each specific type of tissue, such as lung cancer (LC) specimens, before extraction and adjust the laboratory protocol accordingly on a case-by-case basis.

In our study, we compared two modified protocols for extracting DNA from FFPE tissue: one from sections cut directly from the blocks and placed in centrifuge tubes, and the other from sections of glass microscope slides used for histochemical and cytological analysis. Unlike other reports that have conducted comparisons of different extraction methodologies, we provide a summary of our results and lessons learned from our practice in LC, which could be crucial for future FFPE tissue-based studies. Here, we offer details on the laboratory procedures, quantification, and quality control measures of the generated nucleic acid samples. We also offer details regarding the impact of pre-analytical variables on the success of the nucleic acid extraction procedures. Our findings not only enhance the understanding of DNA extraction from FFPE tissue, but also offer practical insights for improving standard operating procedures in future research.

## Material and Methods

### Sample collection

We analyzed 43 samples, consisting of 19 from primary lung tumors (PT) and 24 from mediastinal lymph node (MLN) aspirate obtained through transbronchial needle aspiration guided by echobronchoscopy (EBUS-TBNA) with rapid on-site evaluation (ROSE). All 43 samples underwent two distinct extraction processes (FFPE extraction and slide scraping) as part of the comparative study.

This retrospective study involved samples collected from the Cancer Institute of the State of São Paulo (ICESP - HCFMUSP) between 2011 and 2019. The study was conducted in strict accordance with the Good Clinical Practice and the Declaration of Helsinki principles. It received approval from the appropriate Ethical Committee of Faculdade de Medicina da Universidade de São Paulo, under protocol number 3004 983/2018.

### Extraction of gDNA

We compared the yield of DNA obtained from PT tissues and MLN samples processed and archived in two different ways: 1) sections cut from FFPE and cell blocks, and 2) sections on glass slides with PT tissue stained with hematoxylin and eosin (HE) and slides from aspirated MLN. These MLN samples were obtained by EBUS-TBNA stained with Papanicolaou stain. For both materials, we applied the protocol adapted and validated in our laboratory. As a control reaction, the manufacturer's protocol was strictly followed as a gold standard in paired FFPE samples. We utilized the GeneRead^®^ DNA FFPE Kit (Qiagen GmbH, Germany).

An experienced pathologist previously analyzed all samples to assess the percentage of tumor cells. Only samples with 20% or more tumor cells were included in the study. The EBUS-TBNA cytological smear slides were also evaluated to select slides for scraping based on their cellularity.

The integrity of the gDNA was evaluated by automated capillary electrophoresis using the TapeStation^®^ 2200 (Agilent Technologies, USA). This assessment determined the DNA integrity number (DIN) using a scale from 1 to 10 to measure sample degradation based on the signal distribution across the size of the DNA fragment. The average DIN value obtained was 1.60 (1.65 for PT samples and 1.51 for MLN samples).

#### Sections from FFPE blocks (gold standard protocol)

The manufacturer’s protocol was precisely followed, and the kit is designed for the task at hand (extracting DNA from FFPE tissue). However, we observed low DNA yield and poor DNA integrity, even within the expected range for FFPE tissue. This could be attributed to the prolonged preservation of FFPE tissue under suboptimal environmental conditions, rendering these blocks less suitable as input material for gDNA extraction. Despite being considered the gold standard, the efficacy of the protocol was compromised in this context.

We used a section of 10 µm from each of the paraffin and cell blocks after removing the excess paraffin. Next, 160 uL of deparaffinization solution (organic solvent) was added, vortexed for 10 s, and quickly centrifuged (13,418 *g* for 1 min at 25°C) and incubated at 56°C for 3 min, and then left at room temperature. Then, a mixture comprising 55 µL of RNAse-free water, 25 µL of cell lysis buffer (FTB), and 20 µL of proteinase K was added to the samples. After vortexing and centrifugation for 1 min at 15,093 *g* at 25°C, the samples were incubated for 1 hour at 56°C. Subsequently, the samples were incubated at 90°C for 1 h. The lower clear phase of the sample was transferred to a new 1.5 mL microtube, and 115 uL of RNase-free water was added and vortexed, followed by the addition of 35 µL of the enzyme Uracil-DNA glycosylase (UNG) and incubation at 50°C for 1 h. After incubation, 2 µL of RNAse A was added and thoroughly vortexed.

The purification process began by adding 250 μL of cell lysis buffer (AL, Qiagen). The mixture was vortexed and centrifuged (at 13,418 *g* for 1 min at 25°C). Subsequently, 700 μL of the lysate was transferred to the QIAamp MinElute purification column attached to the collection tube, and centrifuged for 1 min at 13,418 *g* at 25°C. After discarding the material deposited in the collection tube, 500 μL of washing buffer AW1 was added to the column, centrifuged at 13,418 *g* for 1 min at 25°C, and the excess liquid was discarded. This process was repeated with 500 μL of AW2 wash buffer, followed by the addition of 250 μL of ethanol (96-100%) to the columns. Finally, 30 µL of DNA elution buffer (ATE) was pipetted onto the center of the membrane and left for 5 min at room temperature before centrifugation for 1 min at 15,093 *g* at 25°C. The isolated gDNA was then stored at -20°C until quantification.

#### Sections from FFPE blocks (adapted protocol)

In the aforementioned scenario, under the supervision of the Qiagen staff, we made modifications to the Qiagen protocol. This adjustment involved increasing the number of paraffin cuts and omitting the use of the deparaffinization solution provided with the commercial kit. This modification was based on the recommendation of prolonged heat exposure during processing. Four to 6 sections of 10 µm each were cut from the paraffin and cell blocks after removing the excess paraffin. We used 4 slices for larger samples (>4 cm^2^) and 6 slices for smaller samples (<4 cm^2^). Furthermore, a greater number of sections allowed covering more areas of the tumor, which is quite heterogeneous in the case of non-small-cell lung carcinoma, making it relevant for the results.

Next, we added a mixture comprising 55 µL of RNAse-free water, 25 µL of cell lysis buffer (FTB), and 20 µL of proteinase K to the samples. After vortexing and centrifugation for 1 min at 15,093 *g* at 25°C, the samples underwent overnight incubation for 16 h at 56°C. Another important change in the protocol was the prolonged exposure to heat with proteinase K, extending the digestion time with the aim of improving gDNA recovery. Subsequently, the samples were incubated at 90°C for 1 h, followed by the addition of 35 µL of the enzyme Uracil-DNA glycosylase (UNG), and further incubation at 50°C for another hour. After this incubation period, 2 µL of RNAse A was added to the samples and thoroughly vortexed.

The purification process of the adapted protocol was identical to that used in the previously described manufacturer's protocol. At the conclusion of the purification process, 30 µL of DNA elution buffer (ATE) was pipetted onto the center of the membrane and remained for 10 min at room temperature before centrifugation for 1 min at 15,093 *g* at 25°C. Subsequently, the isolated gDNA was stored at -20°C until quantification.

#### Scraping of slides

For this assay, we utilized histological slides from the HE-stained PT samples and cytological slides from Papanicolaou-stained MLN samples. All slides underwent immersion in xylene for 4 to 10 days for the detachment of the coverslips. Once the coverslips were detached, the slides were immersed in 96-100% ethanol, followed by a bath in 70% ethanol, and finally a bath in ultrapure water. Subsequently, after drying, the slides were meticulously scraped using a scalpel and the scraped material was deposited into a 1.5-mL microtube containing 55 µL of RNAse-free water, 25 µL of FTB buffer, and 20 µL of proteinase K. The gDNA extraction and isolation protocol were then followed as previously described.

### Statistical analysis

Sample data and quantifications were tabulated in an Excel spreadsheet and subjected to statistical analysis using SPSS software (IBM SPSS Statistics 25.0, USA). Considering the non-normal distribution of the data, non-parametric tests, such as Mann-Whitney, Kruskal-Wallis (with Bonferroni post-test when applicable), and Wilcoxon tests were employed. Statistical significance was set at P<0.05. We used a logarithmic scale for constructing the graphs and improving the visualization of results.

## Results

### DNA extraction from FFPE blocks

Given the reputable status of the manufacturer and the kit's specific design for extracting DNA from FFPE samples, results suitable for subsequent use in NGS assays were expected with the standard protocol.

Thus, we conducted a pilot assay involving the extraction of genetic material from 18 paired samples, consisting of nine PT samples and nine MLN samples. The PT samples were obtained from surgically resected specimens and biopsies, while all MLN samples were obtained through EBUS-TBNA. As per standardization for assays of this nature, all samples contained 20% or more tumor cells, rendering them suitable for this protocol.

From this standard process, we obtained a median DNA yield of 1.26 ng/µL (range of 0.005 to 8.51 ng/µL) for PT samples and a median DNA yield of 0.04 ng/µL (range of 0.005 to 1.14 ng/µL) for MLN samples (Table 1). Interestingly, despite precisely following the Qiagen gold standard protocol, we observed low DNA yield, even within the expected range for FFPE tissue. This discrepancy may be attributed to the age or quality of the blocks used for gDNA extraction.

**Table 1:** DNA extracted from 18 samples using the gold standard protocol (control) and the adapted protocol.

Samples	Control protocol (ng/μL)	Adapted protocol (ng/μL)
Primary tumor		
1.1	0.005	1.17
7.1	0.962	10.52
8.1	1.31	4.35
9.1	3.28	4.81
11.1	8.51	4.81
15.1	2.65	4.95
22.1	0.121	1.48
24.1	0.986	1.5
26.1	1.26	8.64
Median	1.26	4.81
Mediastinal lymph node		
1.2	0.005	0.049
7.2	1.14	1.31
8.2	0.04	0.045
9.2	0.026	0.065
11.2	0.025	2.5
15.2	0.054	0.162
22.2	0.054	0.094
24.2	0.099	0.005
26.2	0.005	0.053
Median	0.04	0.06

Due to the low yield obtained with the standard protocol, an adapted protocol was used for the same 18 samples. This assay yielded a median DNA of 4.81 ng/µL (range 1.17 to 10.52 ng/µL) for PT samples and a median DNA of 0.06 ng/µL (range 0.005 to 2.50 ng/µL) for MLN samples, as demonstrated in Table 1. The comparison indicated a significanty higher yield with the adapted protocol (P=0.006), as demonstrated in Table 2. Thus, based on these findings, the adapted protocol could be reliably recommended for this type of sample.

**Table 2:** Comparison of gDNA yield between the gold standard protocol and the adapted protocol for formalin-fixed, paraffin-embedded blocks.

Samples	Mean	SD	Median	IQR	Z	P
gDNA gold standard protocol	1.14	2.08	0.11	0.03-1.27	-2.76	0.006
gDNA adapted protocol	2.58	3.15	1.39	0.06-4.81		

SD: standard deviation; IQR: interquartile range. Wilcoxon test; n=18.

### Sections from FFPE blocks *vs* scraping of slides using the adapted protocol

All samples analyzed, including those used in the pilot test, contained a minimum of 20% tumor cells and had been stored for 2 to 9 years (an average of 6 years). For this trial, eight PT samples were obtained from biopsies, while 11 were obtained from surgical resection. All MLN samples were acquired via EBUS-TBNA. The median yield of extracted gDNA from FFPE blocks was 0.18 ng/µL (range 0.005 to 51.00 ng/µL), whereas the median yield of extracted gDNA from slides was 3.11 ng/µL (range 0.153 to 60.00 ng/µL). Differences in DNA yields by storage time, using 5 years as a cutoff point, were compared, but no significant differences were found (P=0.25 for FFPE blocks and P=0.49 for slides).

No significant differences were also found when comparing the quantity of gDNA between PT and MLN samples extracted from FFPE blocks (P=0.08) and scraped from slides (P=0.68) (Table 3).

**Table 3:** Comparison of gDNA yield between FFPE blocks and scrapings from slides.

Samples	Mean	SD	Median	IQR	Z	P
gDNA PT-FFPE	4.79	8.67	0.97	0.11-7.68	-2.99	0.003
gDNA PT-Slide	12.91	18.97	2.82	0.95-18.73		
gDNA MLN-FFPE	2.79	10.14	0.15	0.06-1.28	-3.57	<0.001
gDNA MLN-Slide	12.98	16.41	4.34	2.06-26.52		
gDNA total-FFPE	3.68	9.47	0.21	0.08-2.00	-4.69	<0.001
gDNA total-Slide	12.95	17.41	3.02	1.70-19.25		

SD: standard deviation; IQR: interquartile range; FFPE: formalin-fixed, paraffin-embedded materials; PT: primary lung tumor; MLN: mediastinal lymph node. Wilcoxon test; n=43.

**Figure 1:**
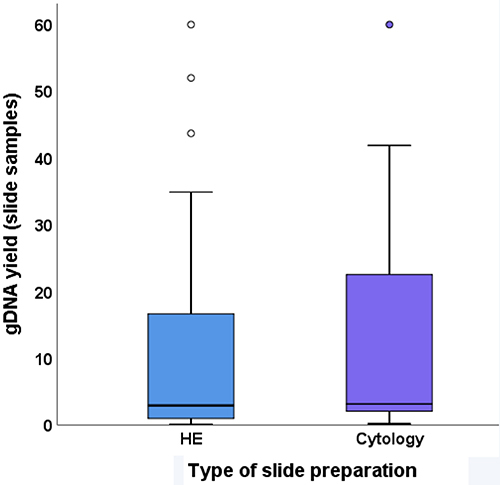
Yield of gDNA extraction from FFPE samples according to the preparation method. Data are reported as median and interquartile range (Mann-Whitney test). HE: hematoxylin and eosin.

We also explored whether the type of slide preparation (HE staining for PT samples and Papanicolaou staining for MLN samples) affected the gDNA yield. The results failed to show a significant difference (P=0.68; Figure 1).

## Discussion

Here, we conducted a study comparing two protocols for extracting gDNA from lung adenocarcinoma tissue and MLN-aspirated FFPE samples. We also utilized protocols for extracting gDNA from different tissue fixation methods. The first protocol involved extracting gDNA directly from sections of FFPE blocks, which were then placed in centrifuge tubes. The second protocol involved extracting gDNA from scraped material fixed on slides for histological and cytological analysis. Our analysis of 43 samples, including 19 from PT and 24 from MLN-aspirated, revealed that we were able to obtain a large yield of gDNA samples with satisfactory quality from most tissues tested.

The results demonstrated that scraping slides with tumor material provided higher yields and better quality of nucleic acids. This observation could be attributed to the smaller surface area of the tissue scraping exposed to the atmosphere, which may protect the nucleic acid from degradation within the cells packed inside the slides (11).

To compare the effectiveness of scrapings from histological and cytological slides with FFPE cuts directly from blocks, the manufacturer's gold standard protocol was precisely followed as a control. This kit is specifically designed to extract and purify DNA from FFPE tissues for sequencing applications, and its optimized protocol suggests using one cut from a paraffin block - a method similar to one of the two approaches we assessed. Surprisingly, we observed low DNA yields when strictly adhering to the kit's recommended procedure. This could be attributed to the long-term preservation of FFPE tissue and poor storage conditions detected at the start. In fact, tissue age poses a challenge for DNA extraction from FFPE tissues. Most of the tissues in our study were more than 5 years old, with samples up to 9 years old.

In the current study, to enhance gDNA yield, we adapted the Qiagen protocol by increasing the number of tissue section cuts and extending the duration of heat exposure to 16 h with proteinase K, instead of solvent-based deparaffinization. This decision was made because heat serves as an alternative to the use of solvents for deparaffinization of samples stored for a long term (more than 3 years). These samples are subject to degradation due to prolonged exposure to different environmental and storage conditions (12). Importantly, FFPE samples should be treated with the UNG enzyme during the degradation process. The process of paraffin fixation and embedding can induce hydrolytic cytosine deamination, resulting in a mutagenic effect by converting cytosine into uracil. This chemical change in the DNA molecule alters base pairs, potentially compromising DNA sequencing. We used UNG in our experimental protocol to repair this chemical change, as demonstrated by previous studies (13,14), and to mitigate the interference in the molecular assays utilizing DNA from FFPE materials.

Other criticisms involving the use of FFPE material are related to the lack of standardization in both the pre-analytical and analytical phases. These inconsistencies can induce chemical alterations and tissue degradation (12,15,16). Such changes result in significant fragmentation and directly affect the yield and quality of gDNA, which is crucial for molecular techniques, like NGS, which demand substantial amounts of high-quality gDNA for satisfactory outcomes. Furthermore, post-fixation conditions, particularly sample storage, temperature, and handling, can also influence the viability and quality of gDNA (17).

In this scenario, we explored alternative sources of material for gDNA extraction in addition to FFPE blocks. Interestingly, the current study demonstrated that gDNA extracted from slide scrapings (histological and/or cytological) can be an excellent alternative, presenting better results and adequate quality for use in molecular techniques, such as NGS (18,19). However, it is important to highlight that the appropriate selection of slides is crucial for the success of gDNA extraction and analyses arising from this material (20-25). Through this modification, and by applying the adjusted method to new sections from the same blocks of previously tested patients, we optimized our approach.

In conclusion, the use of a protocol adapted from the Qiagen GeneRead^®^ DNA FFPE kit was viable for molecular analyses. We recommend slide scrapings as a reliable source of gDNA supply and suggest fine-tuning proteinase K digestion time and heat exposure based on the volume of the initial tumor tissue for optimal results.
